# Rivastigmine Regulates the HIF-1α/VEGF Signaling Pathway to Induce Angiogenesis and Improves the Survival of Random Flaps in Rats

**DOI:** 10.3389/fphar.2021.818907

**Published:** 2022-01-21

**Authors:** Yingying Liu, Wenjie Li, Xinyi Ma, Jibing He, Yi Lin, Dingsheng Lin

**Affiliations:** ^1^ Department of Hand and Plastic Surgery, The Second Affiliated Hospital and Yuying Children’s Hospital of Wenzhou Medical University, Wenzhou, China; ^2^ Second College of Clinical Medical, Wenzhou Medical University, Wenzhou, China

**Keywords:** rivastigmine, HIF-1α/VEGF pathway, angiogenesis, ischemia-reperfusion injury, random skin flap

## Abstract

Random skin flaps are frequently used to repair skin damage. However, the ischemic and hypoxic necrosis limits their wider application. Rivastigmine, a carbamate cholinesterase inhibitor (ChEI), has also been shown to reduce ischemia–reperfusion injury (IRI) and inflammation. This study was performed to examine the effect of rivastigmine on flap survival. Sixty male Sprague–Dawley rats with a modified McFarland flap were randomly divided into three groups: control group, 1 ml of solvent (10% DMSO + 90% corn oil); low-dose rivastigmine group (Riv-L), 1.0 mg/kg; and high-dose rivastigmine group (Riv-H), 2.0 mg/kg. All rats were treated once a day. On day 7, the skin flap survival area was measured. After staining with hematoxylin and eosin (H&E), the pathological changes and microvessel density (MVD) were examined. The expression of inflammatory factors IL-1β and IL-18, CD34, hypoxia-inducible factor-1α (HIF-1α), and vascular endothelial growth factor (VEGF) was examined by immunohistochemical staining. The malondialdehyde (MDA) content and superoxide dismutase (SOD) activity were examined to determine the degree of oxidative stress. Lead oxide/gelatin angiography showed neovascularization and laser Doppler blood flowmetry showed the blood filling volume. Rivastigmine significantly increased the flap survival area and improved neovascularization. CD34, VEGF, and HIF-1α expression were increased, These changes were more pronounced in the Riv-H group. Treatment with rivastigmine reduced the level of MDA, improved SOD activity, and reduced expression of IL-1β and IL-18. Our results indicate that Rivastigmine can increase angiogenesis and significantly improve flap survival.

## Introduction

Skin flaps are widely used for plastic surgery, burns and other applications to repair skin defects caused by tumor resection, trauma, ulcers, large pressure sores, etc., and restore function, correct deformities, and improve appearance(Dehdashtian et al., 2019).There is increasing interest in the function and esthetics of skin flaps for treatment of skin defects(Kushida-Contreras et al., 2021). Random skin flaps, which have no axial blood vessels, are based on the subdermal nerve plexus and rely on capillaries to provide nutrition, are widely used in clinical practice (Kanayama, Mineda et al., 2017; Mao et al., 2019). The size of the available flaps mainly depends on the capillary vascular resistance and perfusion pressure (El Shaer et al., 2019). After the flap is raised, the blood perfusion volume of the distal flap has been reported to decrease to <20% of normal (Roh et al., 2017). Therefore, partial or complete avascular necrosis during flap transplantation is a major problem (Zahir et al., 1998). Based on their histological characteristics, random flaps have a maximum aspect ratio of 1.5–2:1. As the head and neck has relatively abundant microcirculation, the aspect ratio can be increased (Deng et al., 2019). At the same time, the skin flap survival area can also be increased by increasing blood perfusion and constructing a new capillary network. Our previous studies verified that thymosin β4 and azadirachtin A can promote angiogenesis (Lin et al., 2015; He et al., 2020). In addition, inhibition of oxidative stress, inflammation, and pyrolysis can also improve the skin flap survival area (Fang et al., 2020).

Rivastigmine is a carbamate cholinesterase inhibitor (ChEI) with pseudo-irreversible activity that can inhibit not only acetylcholinesterase (AChE), but also butyrylcholinesterase (BuChE) (Enz and Gentsch 2004). Oral administration of rivastigmine was approved by the US Food and Drug Administration (FDA) in 2000 for the treatment of Alzheimer’s disease (AD) (Marucci et al., 2021). The hypothesis of the cause of AD, an important point is that the brain tissue is exposed to oxidative stress and cell damage, and the treatment of rivastigmine can increase the antioxidant capacity, increase glutathione reductase and homocysteine, and reduce the peroxidation product MDA and the inflammatory mediator IL-6 (Gubandru et al., 2013). Rivastigmine can improve dynamic cerebrovascular function by enhancing cholinergic tension (Rosengarten et al., 2009), dilate blood vessels and increase collateral circulation to prevent ischemia, so as to improve Alzheimer’s disease with vascular risk factors (Kumar et al., 2000). Accumulated acetylcholine (ACh) can act on vascular endothelial cells, promote the proliferation of endothelial cells and the production of growth factors such as vascular endothelial growth factor, and have a protective effect on vascular dementia (Wilczynska et al., 2011). The therapeutic effect of rivastigmine on dementia is mainly achieved by inhibiting cholinesterase (ChE) activity and increasing the content and utilization of acetylcholine (ACh) (Lalli and Albanese 2008). ACh has been reported to increase the accumulation of hypoxia-inducible factor-1α (HIF-1α) under normoxic conditions, protect the myocardium from ischemic and hypoxic damage, and exert antiapoptotic effects (Kakinuma et al., 2005). Rivastigmine can restore the sensitivity of blood vessels to ACh and bradykinin, and improve blood circulation (Doganay et al., 2006). Therefore, we know that rivastigmine has a unique effect on regulating angiogenesis, reducing oxidative stress and inflammation response. These effects seem to be beneficial to the survival of the flap.

Angiogenesis is regulated by a number of molecular pathways, including the HIF-1α/VEGF pathway, but there have been few studies regarding regulation by rivastigmine. Based on published studies, we speculate that rivastigmine can increase the content of acetylcholine, which in turn regulates the HIF-1α/VEGF signaling pathway and promotes angiogenesis. Rivastigmine may reduce oxidative stress and inflammation. This study was performed to explore its effects on flap survival and the mechanism of action.

## Material and Methods

### Experimental Animals and Reagents

Healthy adult male Sprague–Dawley rats, aged 6–9 weeks and weighing 200–250 g, were purchased from the Experimental Animal Center of Wenzhou Medical University (WMU) and kept in separate cages under controlled temperature (24°C ± 0.5°C) and humidity (45–50%) conditions, with good ventilation and ad libitum access to water and standard rodent chow. Animal experiments were performed in accordance with the Guidelines for Ethical Review of Laboratory Animals and 3R principles, and were approved by the Ethics Committee for Experimental Animals of Wenzhou Medical University (ID number: WYDW 2017-0509). All experimental procedures were performed using standard methods. Superoxide dismutase (SOD) and malondialdehyde (MDA) detection kits were obtained from Jiangsu Jiancheng Technology Co., Ltd. (Nanjing, Jiangsu, China). Antibodies to IL-1β, IL-18, CD34, VEGF, and HIF-1α were purchased from Affinity Biosciences (Cincinnati, OH, United States). Rivastigmine (≥98% purity) was purchased from Novartis (Basel, Switzerland).

### Animals and Groups

**FIGURE 1:**
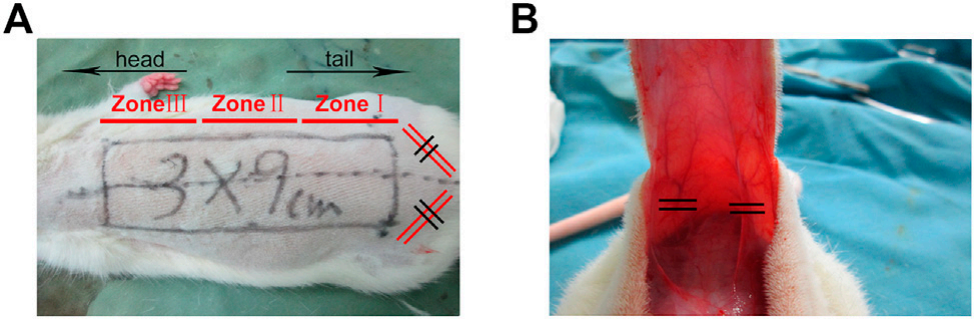
**(A)** Establishment of modified McFarlane skin flap model. The area with length and width of 9 × 3 cm is divided into Zone I, Zone II, and Zone III from the tail to the head. **(B)** The flap was turned over and the iliac blood vessel was disconnected.

Sixty Sprague–Dawley rats were randomized into one of three groups (*n* = 20 each, two rats in each group for pre-experiment, six rats were randomly selected for surgical examination, and paraffin sections were made for H&E staining and Immunohistochemical staining, six rats were used for laser Doppler blood flow detection and lead oxide/gelatin perfusion, and the remaining six rats were used for detection of SOD and MDA in the tissue homogenate): control group, 1 ml of solvent alone; low-dose rivastigmine group (Riv-L), 1.0 mg/kg of rivastigmine; and high-dose rivastigmine group (Riv-H), 2.0 mg/kg rivastigmine. The rats were anesthetized by intraperitoneal (i.p.) injection of 1% sodium pentobarbital (40 mg/kg) and fixed in the prone position. A modified McFarlane flap was made on the back of each rat. Briefly, using the midline of the back of each rat as the axis with a line connecting the iliac crests on both sides as the bottom edge, a 9 cm × 3 cm ultralong random skin flap was made. Maintaining the subdermal capillary network, the two iliac arteries were separated and ligated. Bleeding spots on the wound surface were ligated during the experiment. After lifting the skin flap completely, it was immediately closed *in situ* using a 4-0 blunt-tipped suture needle (Figure 1). Sterility was maintained throughout the surgical process, and erythromycin ointment was applied to the wound after the operation to prevent infection. To prevent self-injury after the operation, headgear designed by members of the research group was applied to the rats and they were kept in separate cages. To reduce interoperator variation, all surgical operations were performed by the same operator. 50 mg of Rivastigmine was formulated into a solution with a concentration of 0.25 mg/ml in a solvent composed of 10% DMSO and 90% corn oil, in accordance with the supplier’s instructions. With reference to the drug manufacturer’s instructions and a previous study by Kumar et al., (Kumar and Kumar 2009) the animals were given rivastigmine by gavage at a dose of 1.0 mg/kg once a day in the Riv-L group and 2.0 mg/kg once per day in the Riv-H group. The control group was given 1 ml of solvent composed of 10% DMSO and 90% corn oil.

### Morphological Observations and Assessment of Skin Flap Survival Area

Each flap was divided into three equal parts designated (areas I–III) from tail to head. The general condition of the rat skin flaps, including color, texture, range of necrosis, hair growth, etc., was examined every day after the operation. All rats were euthanized after 7 days of drug treatment. Cellophane was used to carefully outline the surviving and the areas of the skin flaps, and they were then cut out and weighed using an electronic scale. The percentage of skin flap survival area was calculated as follows: skin flap survival area cellophane mass/skin flap total surface area cellophane mass × 100%. Each rat was assessed twice, and the average value was taken. The surviving area was pink with a soft texture and new hair growth, while the necrotic area was dark brown, crusted, hard in texture, and free from hair growth.

### Determination of SOD and MDA Levels

On day 7, six rats were selected at random from each group to obtain tissue samples. A flap tissue sample measuring 0.5 cm × 0.5 cm was separated from the center of zone II; the muscle layer was removed, and the tissue was placed in an ice water bath. The homogenate was then centrifuged and the supernatant was retained. Xanthine oxidase was used to determine the SOD activity, and the thiobarbituric acid (TBA) method was used to determine the MDA content.

### Histopathological Evaluation

On day 7, a 1.0 cm × 1.0 cm sample of tissue was obtained from zone II of each flap. The specimen was immersed in 4% paraformaldehyde, and after approximately 24 h the tissue was completely wrapped in paraffin and cut into sections 4-μm-thick sections for detection of histological indicators. The paraffin sections were stained with hematoxylin and eosin (H&E). The histological characteristics of the flaps were examined under an optical microscope. Skin flap capillary angiogenesis, neutrophil infiltration, tissue edema, granulation tissue thickness, etc. were assessed. The degree of neutrophil infiltration was evaluated under an optical microscope at ×100 magnification. The number of new blood vessels was counted in six randomly selected high-power fields (200 ). Then, we counted the number of microvessels per unit area (/mm^2^), which was taken as the microvessel density (MVD).

### Immunohistochemical Determination of HIF-1α, VEGF, CD34, IL-1β, IL-18 Expression

The prepared paraffin sections were subjected to immunohistochemical staining using the streptavidin-peroxidase method. Under a microscope, positive staining appeared as brownish-yellow particles. The integrated absorbance (IA) of the positive particles of each flap tissue was measured, and averages were considered as the relative expression of IL-1β, IL-18, CD34, VEGF, and HIF-1α. Photographs were taken of six randomly selected high-power fields (200×) and imported into Image-Pro Plus v6.0 (Media Cybernetics, Bethesda, MD, United States) to calculate the levels of the above molecules.

### Laser Doppler Flowmetry to Determine Blood Perfusion Volume

On day 7, six rats selected at random from each of the three groups were anesthetized and placed in a rectangular area delineated by the laser Doppler probe, and the blood flow in each area of the flap (zones I–III) was measured. The blood perfusion unit (BPU) is an index of blood perfusion calculated as the flow rate multiplied by the red blood cell concentration. Color Doppler images of the skin flaps were analyzed using MoorLDI Review V6.1 software (Moor Instruments Ltd., Axminster, United Kingdom).

### Lead Oxide/gelatin X-Ray Angiography

On day 7, six rats were selected at random from each of the three groups for intravascular infusion of lead oxide/gelatin and X-ray angiography. The neck was dissected and the blood vessels were carefully separated. Arterial cannulation was performed with a heparinized 22 G catheter and used for perfusion with isotonic saline at 37°C, with drainage from the ipsilateral jugular vein. After the drainage fluid had clarified, lead oxide/gelatin contrast agent (100 ml/kg) was injected, taking care to avoid vasospasm throughout the process. After perfusion, the specimens were refrigerated and X-ray imaging was performed on the following day.

### Statistical Analysis

All experimental data are expressed as the mean ± standard deviation (SD). One-way analysis of variance (ANOVA) was used to determine the significance of differences between groups with *p* < 0.05 taken to indicate statistical significance. All statistical analyses were performed using IBM SPSS 25.0 (IBM Corp., Armonk, NY, United States).

## Results

### Rivastigmine Significantly Increased Flap Survival Area

**FIGURE 2:**
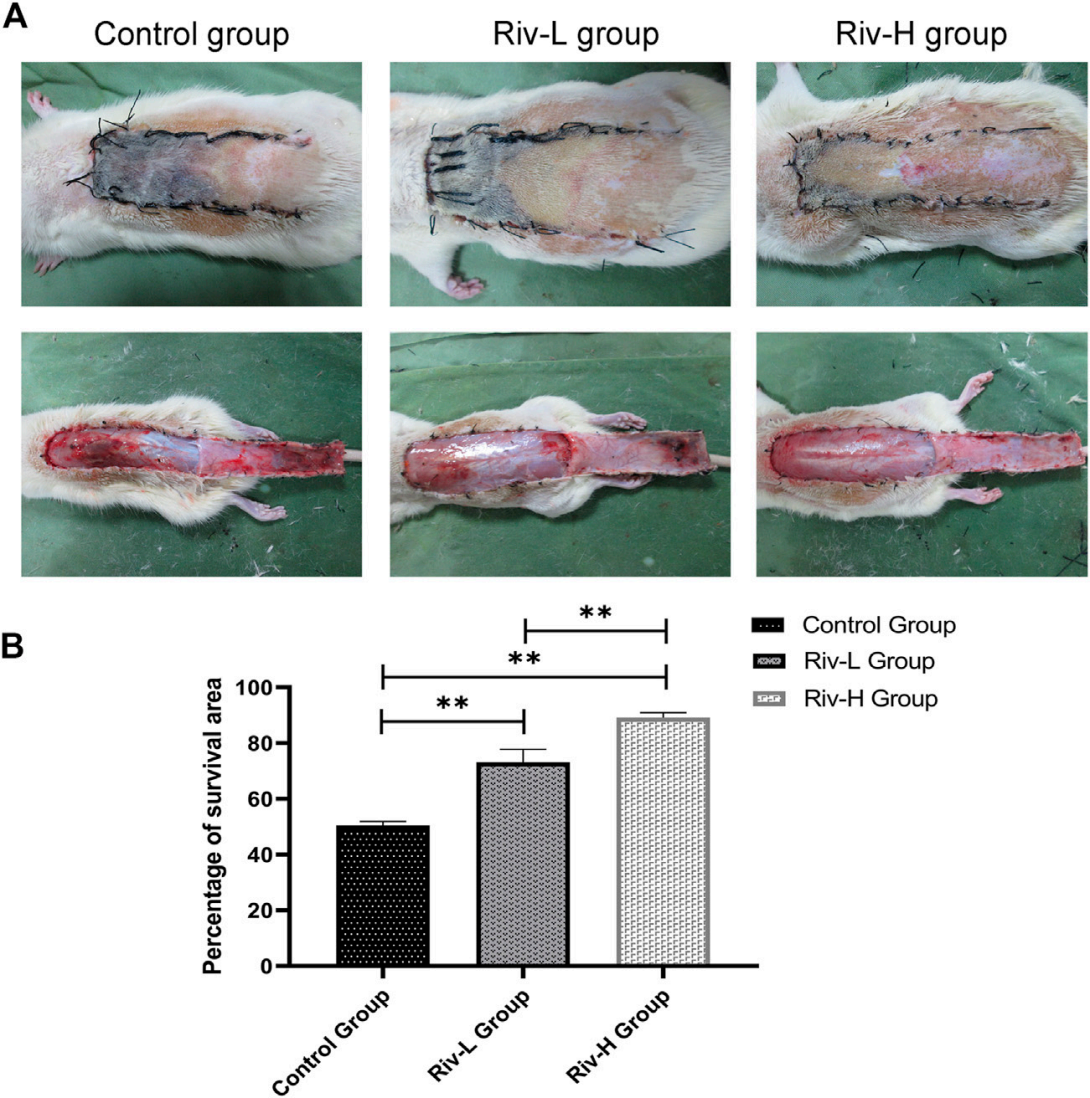
**(A)** Macroscopic observation of the random flap on the back of a rat on day 7 after surgery (external and after the flap has been lifted). Necrotic skin flaps become burnt black and rough, and the surviving skin flaps are soft and have hair growth. **(B)** Comparison of flap survival rate among the three groups. **p < 0.01.

Necrosis of the distal flap tissue progressed after completion of the operation, and there was a very clear boundary between the necrotic and surviving areas on day 7. The necrotic tissue was black in color and hard in texture, with an uneven surface and no hair growth or bleeding of the incised tissue (Figure 2). The skin flaps in the surviving area were light red in color, with a soft texture and dense hair growth. Inspection of the skin flap revealed an interlaced capillary network in the surviving area (Figure 2).

Macroscopic observation revealed severe necrosis in the control group. The total area of skin flap necrosis was significantly larger in the control than rivastigmine groups. The control group showed significantly lower flap survival rate (50.44 ± 1.30%) than the Riv-L group (70.12 ± 4.30%) and Riv-H group (89.16 ± 1.60%) (both *p* < 0.01).

### Histological Observation of the Effects of Rivastigmine Application on Neutrophil Infiltration and MVD

**FIGURE 3:**
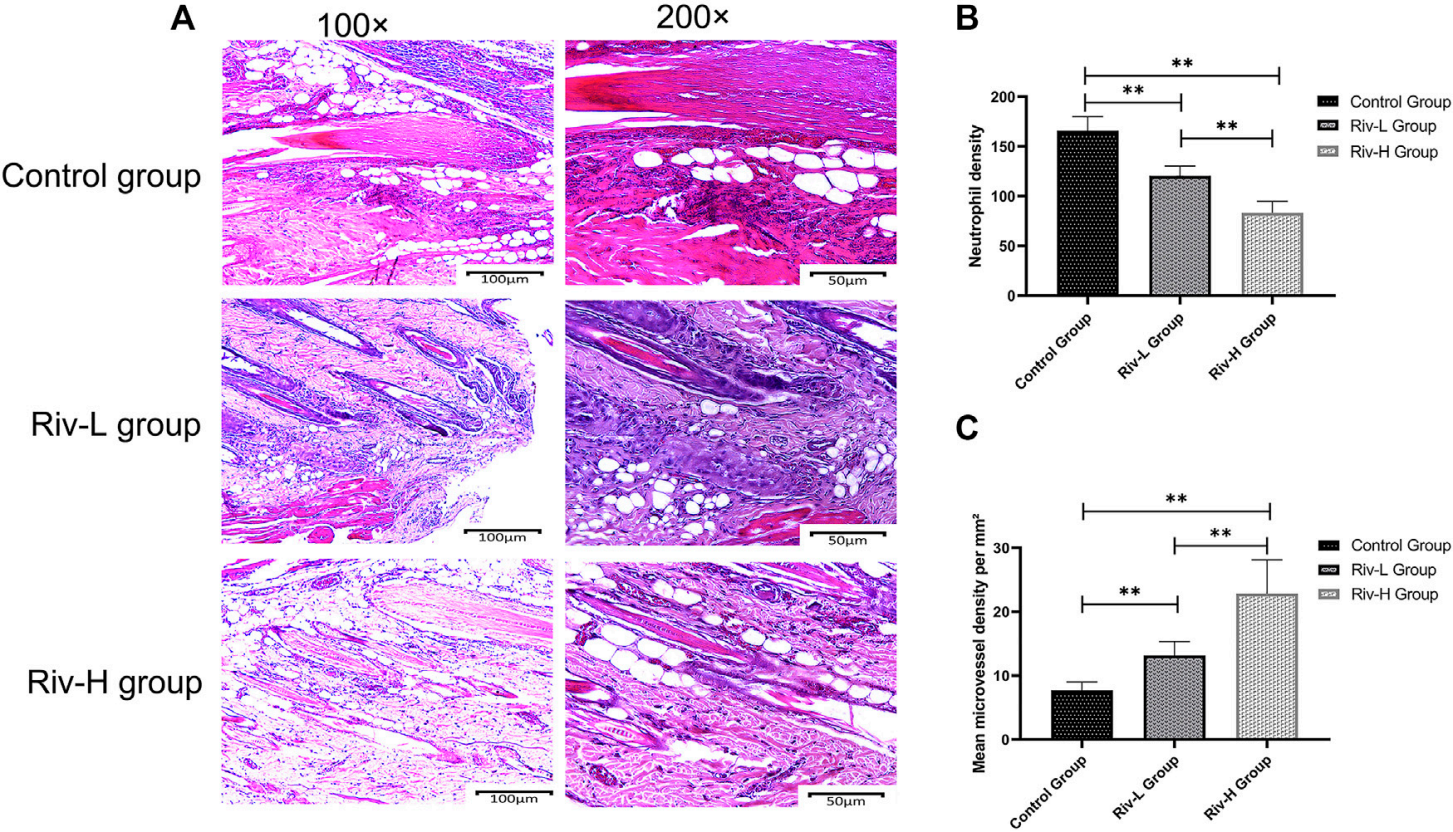
**(A)** Pathological characteristics of H&E staining of random skin flaps in zone II tissue of the three groups revealed by optical microscopy (×100 and ×200 magnification). **(B)** Comparison of neutrophil density among the skin flaps of the three groups. **(C)** Comparison of microvessel density (MVD) among the skin flaps of the three groups. The rivastigmine treatment groups showed significantly increased MVD compared to the control group. **p < 0.01.

The H&E-stained paraffin sections were observed under an optical microscope. In each group, ischemic skin flap zone II had varying degrees of inflammatory changes, i.e., tissue edema, infiltration of large numbers of neutrophils, and fibrous hyperplasia (Figure 3A). The neutrophil infiltration density of Riv-H was 83.16 ± 10.53/mm^2^, which was significantly lower compared to both the Riv-L group (120.35 ± 9.07/mm^2^, *p* < 0.01) and control group (165.79 ± 12.93/mm^2^, *p* < 0.01) (Figure 3B). The MVD determined by light microscopy (×200 magnification) was significantly higher in the Riv-H and Riv-L groups in comparison to the control group (22.80 ± 4.85/mm^2^, 13.16 ± 2.00/mm^2^, 7.72 ± 1.16/mm^2^, respectively, *p* < 0.01) (Figure 3C).

### Rivastigmine Markedly Reduced Oxidative Stress

**FIGURE 4:**
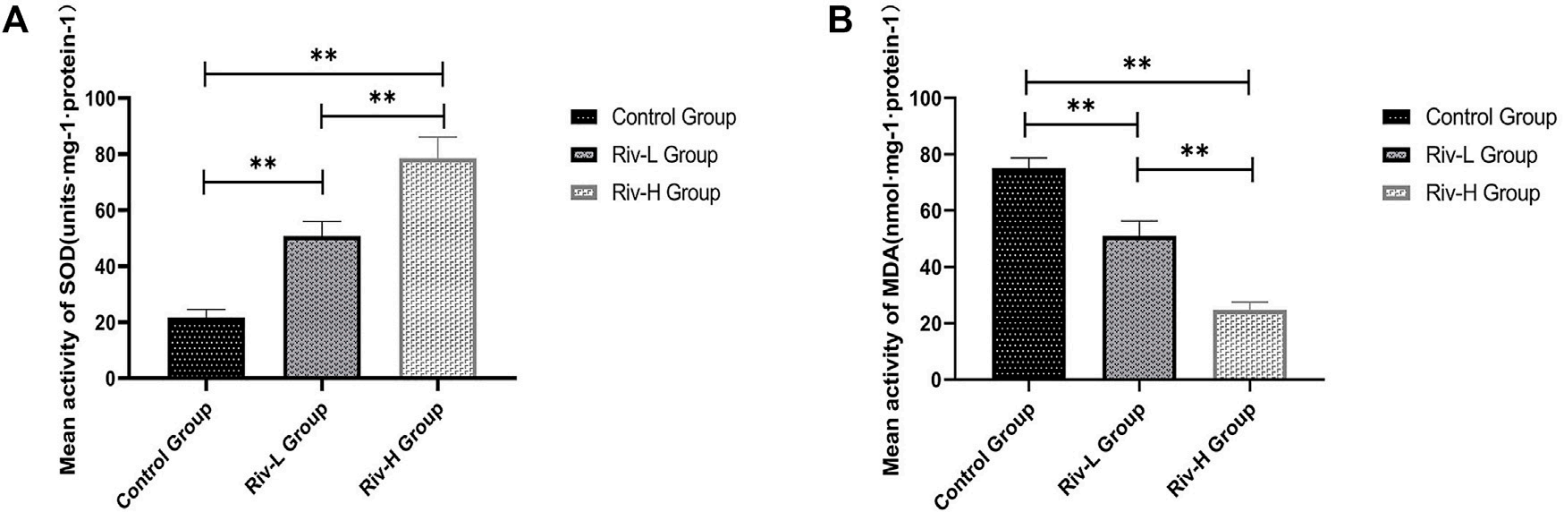
**(A)** Average SOD activities in rat skin flap tissue. **(B)** Comparison of average MDA contents in rat skin flap tissue among groups. Rivastigmine reduced oxidative stress. Compared to the control group, low and high doses of rivastigmine resulted in significant changes in SOD and MDA. There were also significant differences between the Riv-L and Riv-H groups. **p < 0.01.

SOD and MDA are commonly used indicators of oxidative stress. The average SOD activities of the control, Riv-L, and Riv-H groups were 21.73 ± 2.59, 50.74 ± 4.73, and 78.57 ± 7.00 U mg protein^−1^, respectively, indicating that rivastigmine treatment significantly increased the SOD activity (*p* < 0.01) (Figure 4A). The average MDA contents of the Riv-L and Riv-H groups (50.92 ± 4.93 and 24.70 ± 2.33 U·mg protein, respectively) were significantly lower compared to the control group (75.16 ± 3.28 U·mg protein, *p* < 0.01) (Figure 4B).

### Rivastigmine Reduced Inflammatory Cytokine Expression

**FIGURE 5:**
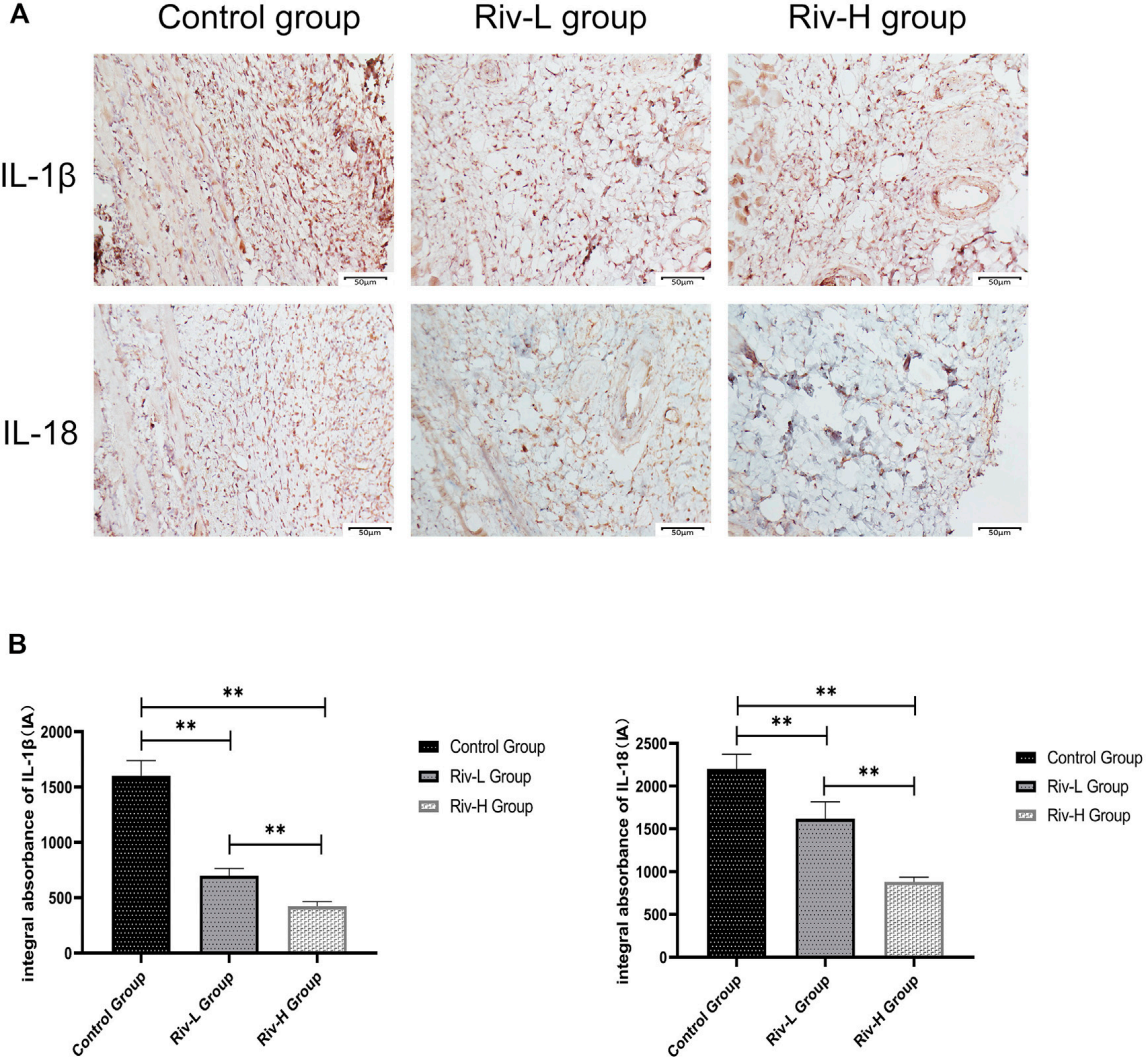
**(A)** The tissues in area II of the flaps in the three groups were subjected to immunohistochemical analysis to examine IL-1β and IL-18 expression (×200 magnification) **(B)** The levels of IL-1β and IL-18 expression were quantified by measuring IA. **p < 0.01.

Immunohistochemical staining was performed to measure the expression of proinflammatory cytokines in area II of ischemic skin flaps (Figure 5A). The levels of IL-1β expression were lower in the Riv-H and Riv-L groups (422.17 ± 39.04 and 699.83 ± 59.00 IA, respectively) than the control group (1,601.50 ± 126.33 IA). In addition, the Riv-H and Riv-L groups showed significantly lower levels of IL-18 expression than the control group (879.67 ± 49.45, 1,618.83 ± 180.28, and 2,201.67 ± 154.66 IA, respectively (Figure 5B).

### Rivastigmine Enhanced Perfusion in the Skin Flaps

**FIGURE 6:**
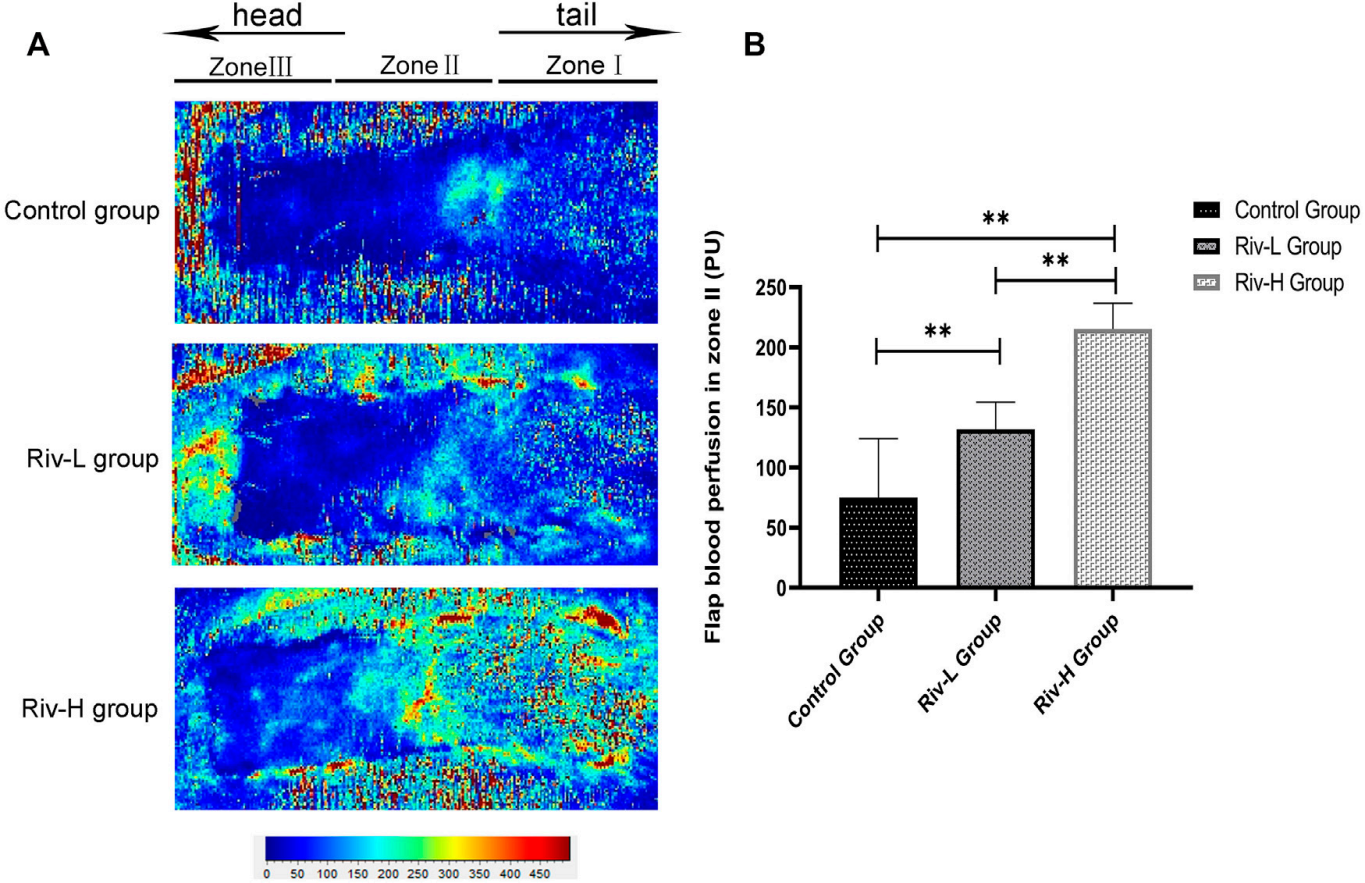
**(A)** Laser Doppler detection showed blood perfusion in the flaps. **(B)** The blood filling volume of the flap tissue (BPU). The differences between the rivastigmine treatment groups and control group were significant. **p < 0.01.

Laser Doppler blood flow imaging (Figure 6A) indicated improved blood perfusion in the flaps in the Riv-L and Riv-H groups (132.02 ± 20.49 and 215.32 ± 19.37 BPU, respectively) compared to the control group (75.02 ± 44.86 BPU, *p* <0.01) (Figure 6B).

### Rivastigmine Improved the Microcirculation of the Distal Flap

**FIGURE 7:**
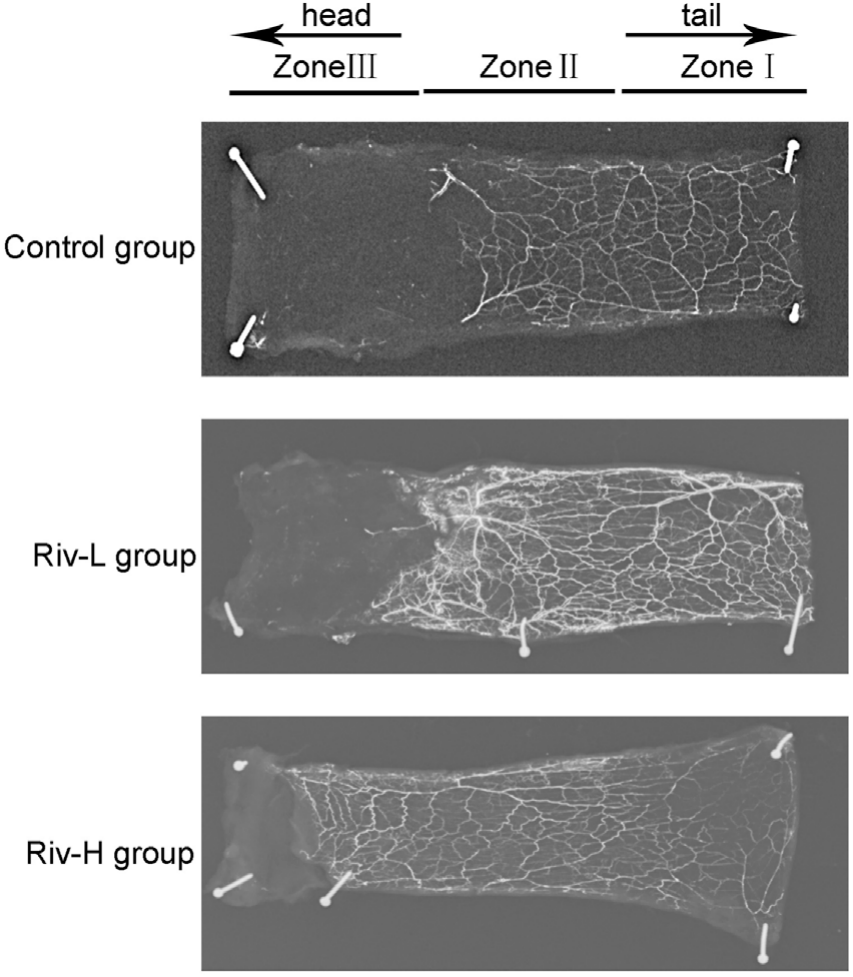
Lead oxide dissolved in gelatin was injected into the blood vessels of rats for X-ray imaging. Rivastigmine significantly enhanced construction of the capillary network. **p < 0.01.

X-ray angiography revealed that the Riv-H and Riv-L groups had more new blood vessels and vascular anastomosis than the control group, suggesting that rivastigmine improves the blood microcirculation of the distal flap (Figure 7).

### Rivastigmine Increased CD34, VEGF, and HIF-1α Expression

**FIGURE 8:**
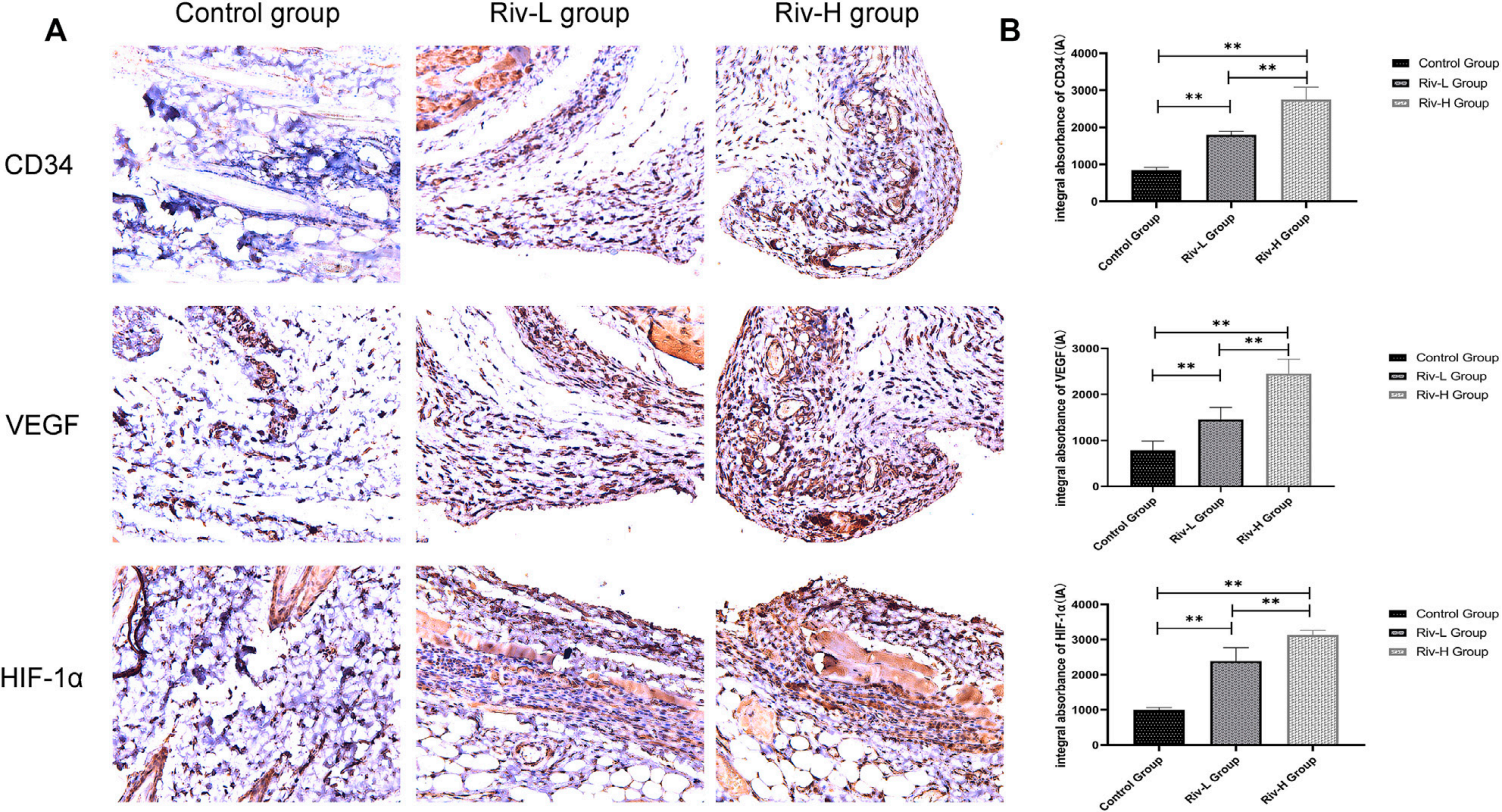
**(A)** CD34, VEGF and HIF-1α expression were measured in the skin flaps of the three groups by immunohistochemical analysis. **(B)** CD34, VEGF and HIF-1α expression levels in the three groups. Rivastigmine treatment increased the expression of CD34, VEGF and HIF-1α. **p < 0.01.

Consistent with our hypothesis, (Figure 8A) the Riv-H and Riv-L groups had higher HIF-1α contents in flap tissues (3132.83 ± 120.10 and 2385.83 ± 348.72 IA, respectively) compared to the control group (1003.17 ± 58.57 IA) (Figure 8B). The levels of VEGF expression were significantly higher in the Riv-H and Riv-L groups than the control group (2,450.50 ± 288.81, 1,456.67 ± 238.38, and 788.67 ± 181.24 IA, respectively, *p* < 0.01) (Figures 6B, 8B), indicating that rivastigmine can significantly increase the level of VEGF in ischemic skin flaps to promote blood vessel generation, consistent with the angiography results presented above. In addition, the level of CD34 expression was significantly higher in the Riv-H and Riv-L groups (2,750.67 ± 304.44 IA and 1,797.67 ± 91.65 IA) than the control group (843.67 ± 71.29 IA, *p* < 0.01) (Figure 8B). The difference between the high- and low-dose groups was also significant (*p* < 0.01).

## Discussion

Skin flaps do not have a complex composition, comprising only skin and subcutaneous fat tissue. They can be separated from intact skin and then transplanted onto superficial areas with defects or deformities (Mao et al., 2019). As the flaps do not contain major blood vessels, the supply of capillaries on which they depend is limited. Necrosis of the distal flap is the most serious and common complication, affecting the function and appearance of organs, limiting clinical application, prolonging the hospital stay, and increasing both the economic burden and psychological pressure on the patient (Baris et al., 2013). Therefore, there has been a great deal of research directed toward new therapeutic drugs and surgical options. Studies have shown that skin flaps are not only ischemic, but also in a state of blood congestion after elevation (Kanayama et al., 2017). Increasing the formation of new vessels and improving the blood supply can increase the likelihood of distal flap survival.

In contrast to other ChEIs, rivastigmine inhibits not only AChE, but also BuChE. Rivastigmine inhibits the activity of ChE and reduces the decomposition of ACh, and has been used to relieve the symptoms of dementia, Huntington’s disease (Kumar and Kumar 2009), and cognitive impairment in patients with multiple sclerosis (Mäurer et al., 2013).Furthermore, rivastigmine is beneficial to vascular dementia (Román 2005) or complicated vascular diseases and increases cerebral blood flow (CBF). We found that the effects of rivastigmine on angiogenesis and blood circulation seem to be related to the regulation of HIF-1α and VEGF.

HIF-1, the key molecule in the regulation of oxygen homeostasis, is a vital nuclear transcription regulator that promotes angiogenesis and is therefore an important target for various therapeutic measures. HIF-1 is a dimer composed of two different subunits, i.e., the HIF-1α subunit regulated by oxygen concentration and HIF-1β nuclear subunit (which is continuously expressed) (Shafighi et al., 2011). The level of HIF-1 transcription is regulated by the content of HIF-1α, which changes dynamically in response to the oxygen concentration. Under normal non-hypoxic conditions, the proline residues on the HIF-1α subunit are recognized and bound by prolyl hydroxylases (PHDs), and the hydroxylated HIF-1α binds to Von Hippel-Lindau (VHL) protein and undergoes degradation in the proteasome through VHL-mediated E3 ubiquitin ligase recognition (Patel et al., 2005; Qiu et al., 2019). However, under hypoxic conditions, hydroxylation by PHDs is inhibited, leading to an accumulation of HIF-1α, which binds to HIF-1β. The dimer then migrates to the nucleus and interacts with the hypoxia-response elements (HREs) in the promoters of downstream oxygen-sensitive genes, such as VEGF, PDGF, angiogenin, (Patel et al., 2005) etc., inducing their transcription (Cury et al., 2013; Wang et al., 2014). VEGF is one of the most critical proangiogenesis regulators, which promotes the expression, division, and migration of endothelial cells to increase angiogenesis (Lv et al., 2018). VEGF can also improve the permeability of blood vessels and increase blood flow. CD34 is not only a surface marker of hematopoietic progenitor cells, but is also expressed on the surface of endothelial cells, which is related to the formation of new blood vessels (Peichev et al., 2000).

Kakinuma et al. reported that ChEIs increased HIF-1α and VEGF expression in an ischemic hind limb model, and promoted angiogenesis through activation of the HIF-1α/VEGF signaling pathway (Kakinuma et al., 2010). Hao et al. also confirmed that ChEIs can improve hypoxia-induced angiogenesis through the regulation of the HIF-1α/VEGF pathway (Hao et al., 2014). Previous studies showed that ACh upregulates HIF-1α, induces the expression of VEGF, and promotes angiogenesis to ameliorate the destructive effects of ischemia and hypoxia on cardiomyocytes (Kakinuma et al., 2013). Rivastigmine has also been shown to promote angiogenesis and reduce endothelial cell apoptosis (Mortazavian et al., 2013). These studies suggested that the mechanism by which rivastigmine promotes the survival of skin flaps may be related to increased ACh activity, for regulation of the HIF-1α/VEGF pathway. In our present study, immunohistochemical analysis on day 7 after surgery showed that rivastigmine treatment upregulated the expression of HIF-1α, VEGF, and CD34 in a dose-dependent manner. Histopathological analysis indicated that rivastigmine treatment increased the MVD. Moreover, laser Doppler flowmetry and lead oxide/gelatin contrast-enhanced X-ray imaging showed that rivastigmine promoted angiogenesis in the distal flap, increased blood perfusion, and improved blood circulation. Minimizing the time required for establishing blood circulation and increasing the blood filling volume in the distal flap can increase the likelihood of flap survival.

IRI is a complex pathophysiological process that occurs as a complication of skin flap necrosis. Current research suggests that the production of reactive oxygen species (ROS), energy metabolism disorders, vascular endothelial cell dysfunction, etc., are important mechanisms underlying flap IRI, (Ju et al., 2020) with ROS expression being the most important factor. The accumulation of ROS exacerbates the inflammatory response of the tissue. Therefore, the two processes of IRI and inflammatory reaction form a vicious circle, exacerbating the necrosis of flap tissue. MDA, as the end product of the oxidation reaction of ROS with biological membrane lipids, directly reflects the extent of membrane lipid peroxidation. SOD is an important antioxidant metalloenzyme that removes superoxide anions through disproportionation, and maintains the dynamic balance of oxidation and antioxidant reactions. Published studies have proved that the treatment of rivastigmine can reduce oxidative stress and inflammation (Ali et al., 2018), increase SOD levels, and reduce MDA (Kumar and Kumar 2009) through different rat models. Rivastigmine also reduces the inflammatory cytokines TNF-α, IL-6 and CRP (Akhtar et al., 2020). The study by Chies A et al. showed that rivastigmine pretreatment can reduce the liver injury induced by ischemia-reperfusion in rats, and proposed that this effect seems to be related to the cholinergic anti-inflammatory pathway (Chies et al., 2018). The cholinergic anti-inflammatory pathway relies on ACh from neural or non-neural sources to exert central and peripheral anti-inflammatory effects and reduce the release of inflammatory cytokines (Bonaz et al., 2016; Han et al., 2017). It can promote the healing process of mice skin wounds, regulate the proliferation of vascular endothelial cells and mediate angiogenesis (Fan et al., 2011). In our preliminary study, we found that rivastigmine treatment can significantly reduce the level of oxidative stress, that is, SOD activity is increased, MDA level is reduced, and the level of pro-inflammatory cytokines is also reduced. But whether the anti-inflammatory effect of rivastigmine in this experiment is related to the cholinergic anti-inflammatory pathway, our experiment still cannot explain, but we may try to explore this mechanism next.

Up to now, there has been very little research on rivastigmine outside the field of neurology. Our research is currently only basic animal experiments. Whether it can be further applied to human skin flap transplantation requires further experiments to verify.

## Conclusion

The results of this study confirmed that rivastigmine upregulates the expression of HIF-1α and VEGF, and regulates the HIF-1α/VEGF signaling pathway. Rivastigmine significantly promoted angiogenesis and improved the survival of skin flaps. In addition, rivastigmine reduced oxidative stress and inflammation. Therefore, rivastigmine is a promising drug for use in skin flap transplantation.

## Data Availability

The original contributions presented in the study are included in the article/Supplementary Material, further inquiries can be directed to the corresponding author.
